# Draft Genome Sequence of *Microbacterium* sp. Strain UCD-TDU (Phylum *Actinobacteria*)

**DOI:** 10.1128/genomeA.00120-13

**Published:** 2013-03-21

**Authors:** Zachary A. Bendiks, Jenna M. Lang, Aaron E. Darling, Jonathan A. Eisen, David A. Coil

**Affiliations:** University of California at Davis, Genome Center, Davis, California, USA

## Abstract

Here, we present the draft genome sequence of *Microbacterium* sp. strain UCD-TDU, a member of the phylum *Actinobacteria*. The assembly contains 3,746,321 bp (in 8 scaffolds). This strain was isolated from a residential toilet as part of an undergraduate student research project to sequence reference genomes of microbes from the built environment.

## GENOME ANNOUNCEMENT

Organisms within the genus *Microbacterium* are aerobic, rod-shaped, Gram-positive bacteria and have been isolated from numerous environments, including human clinical patients (1), pork sausage (2), dairy products and production equipment (3), nematodes (4), and radioactive sites (5).

*Microbacterium* sp. strain UCD-TDU was isolated from a toilet in Davis, California. Scrapings were incubated overnight in Luria broth (LB) at 37°C and plated on LB agar. Single colonies were picked for serial dilution streaking and were identified by Sanger sequencing of the 16S rRNA gene after PCR amplification (using primers 1391R and 27F). Genomic DNA was extracted from fresh overnight cultures using a Wizard genomic DNA purification kit (Promega). Illumina paired-end libraries were then made from sonicated DNA using a TruSeq DNA sample prep v2 kit (Illumina). Fragments between 300 and 600 bp were selected using a Pippin Prep DNA size selection system (Sage Science).

A total of 2,370,532 paired-end reads were generated on an Illumina MiSeq at a read length of 160 bp. Quality trimming and error correction of the reads resulted in 2,263,498 high-quality reads that produced an overall coverage of ~97×. All sequence processing and assemblies were performed using the a5 assembly pipeline (6). This pipeline automates the processes of data cleaning, error correction, contig assembly, scaffolding, and quality control. The total length of the assembled *Microbacterium* sp. UCD-TDU genome is 3,746,321 bp, with a G+C content of 68.36%. The assembly is contained in 8 scaffolds with a mean scaffold size of 468,290 bp and an N_50_ of 1,056,891 bp. During scaffolding, some contigs were merged based on short overlaps and read pair information, yielding a final collection of 15 contigs in 8 scaffolds.

Gene annotation was performed with the Rapid Annotations using Subsystems Technology (RAST) annotation server (7). *Microbacterium* sp. UCD-TDU contains 3,667 predicted protein-coding sequences and 52 predicted noncoding RNAs. A full-length (1,488 bp) 16S sequence was obtained from this annotation and was used to assign taxonomy to our isolate. A phylogenetic tree of 200 cultured isolates of *Microbacterium* species was produced using the Ribosomal Database Project (RDP), which implements a weighted neighbor-joining tree building algorithm (8). *Microbacterium* sp. UCD-TDU falls within a poorly resolved paraphyletic clade, containing primarily *Microbacterium oxydans*, as well as several other species of *Microbacterium* (doi:10.6084/m9.figshare.628065). Because the 16S rRNA gene sequence of *Microbacterium* sp. UCD-TDU has >99% identity with those of several other *Microbacterium* species and the phylogenetic relationships within the genus are unclear, we are unable to assign a species name to this isolate. Completeness of the genome was assessed using the Phylosift software (A. Darling, G. Jospin, E. Lowe, E. Matsen, H. Bik, and J. Eisen, unpublished data), which searches for a list of 40 highly conserved single-copy marker genes (D. Wu, G. Jospin, and J. Eisen, unpublished data), and all 40 were found in this assembly.

Several other genomes from *Microbacterium* have been sequenced, including *M. barkeri* (9), *M. yannicii* (10), *M. laevaniformans* (11), and *M. testaceum* (12). The 16S rRNA gene of *Microbacterium* sp. UCD-TDU has a high identity with these other species (95%, 97%, 97%, and 98%, respectively), but none of them are found in the *M. oxydans-*dominated clade described above.

### Nucleotide sequence accession numbers. 

The draft genome sequence for *Microbacterium* sp. UCD-TDU has been included in the GenBank Whole-Genome Shotgun (WGS) database under the accession no. AOSO00000000. The version described in this paper is the first version, accession no. AOSO01000000. Illumina reads are available at http://dx.doi.org/10.6084/m9.figshare.157179.

## References

[1] Funke G Falsen E Barreau C 1995 Primary identification of *Microbacterium* spp. encountered in clinical specimens as CDC coryneform group A-4 and A-5 bacteria. J. Clin. Microbiol. 33:188–192 7699039 10.1128/jcm.33.1.188-192.1995 PMC227905

[2] McLean RA Sulzbacher WL 1953 *Microbacterium thermosphactum*, spec. nov.; a nonheat resistant bacterium from fresh pork sausage. J. Bacteriol. 65:428–433 13069397 10.1128/jb.65.4.428-433.1953 PMC169546

[3] Nashif SA Nelson FE 1953 Some studies on microbacteria from Iowa dairy products. Appl. Microbiol. 1:47–52 13008430 10.1128/am.1.1.47-52.1953 PMC1056857

[4] Hodgkin J Kuwabara PE Corneliussen B 2000 A novel bacterial pathogen, *Microbacterium nematophilum*, induces morphological change in the nematode C. elegans. Curr. Biol. 10:1615–1618 11137017 10.1016/s0960-9822(00)00867-8

[5] Nedelkova M Merroun ML Rossberg A Hennig C Selenska-Pobell S 2007 *Microbacterium* isolates from the vicinity of a radioactive waste depository and their interactions with uranium. FEMS Microbiol. Ecol. 59:694–705 17381522 10.1111/j.1574-6941.2006.00261.x

[6] Tritt A Eisen JA Facciotti MT Darling AE 2012 An integrated pipeline for *de novo* assembly of microbial genomes. PLoS One 7:e42304 23028432 10.1371/journal.pone.0042304 PMC3441570

[7] Aziz RK Bartels D Best AA DeJongh M Disz T Edwards RA Formsma K Gerdes S Glass EM Kubal M Meyer F Olsen GJ Olson R Osterman AL Overbeek RA McNeil LK Paarmann D Paczian T Parrello B Pusch GD Reich C Stevens R Vassieva O Vonstein V Wilke A Zagnitko O 2008 The RAST server: rapid annotations using subsystems technology. BMC Genomics 9:75 18261238 10.1186/1471-2164-9-75 PMC2265698

[8] Cole JR Wang Q Cardenas E Fish J Chai B Farris RJ Kulam-Syed-Mohideen AS McGarrell DM Marsh T Garrity GM Tiedje JM 2009 The Ribosomal Database Project: improved alignments and new tools for rRNA analysis. Nucleic Acids Res. 37:D141–D145 19004872 10.1093/nar/gkn879 PMC2686447

[9] Liu J Zhou Q Ibrahim M Liu H Jin G Zhu B Xie G 2012 Genome sequence of the biocontrol agent *Microbacterium barkeri* strain 2011-R4. J. Bacteriol. 194:6666–6667 23144410 10.1128/JB.01468-12 PMC3497549

[10] Sharma P Diene SM Gimenez G Robert C Rolain J-M 2012 Genome sequence of *Microbacterium yannicii*, a bacterium isolated from a cystic fibrosis patient. J. Bacteriol. 194:4785 22887680 10.1128/JB.01088-12 PMC3415485

[11] Brown SD Palumbo AV Panikov N Ariyawansa T Klingeman DM Johnson CM Land ML Utturkar SM Epstein SS 2012 Draft genome sequence for *Microbacterium laevaniformans* strain OR221, a bacterium tolerant to metals, nitrate, and low pH. J. Bacteriol. 194:3279–3280 22628508 10.1128/JB.00474-12 PMC3370866

[12] Morohoshi T Wang WZ Someya N Ikeda T 2011 Genome sequence of *Microbacterium testaceum* StLB037, an *N*-acylhomoserine lactone-degrading bacterium isolated from potato leaves. J. Bacteriol. 193:2072–2073 21357489 10.1128/JB.00180-11 PMC3133038

